# The Effect of Pregabalin on Microglia Differentiation in Rat with Neuropathic pain: A Preliminary Study

**DOI:** 10.7150/ijms.96236

**Published:** 2024-05-13

**Authors:** Seung-Wan Hong, Liyun Piao, Eun-Hwa Cho, Eun-Hye Seo, Seong-Hyop Kim

**Affiliations:** 1 Department of Anesthesiology and Pain medicine, Konkuk University Medical Center, Konkuk University School of Medicine, Seoul, Korea.; 2 Department of Infection and Immunology, Konkuk University School of Medicine, Seoul, Korea.; 3 Korea mRNA vaccine initiative, Gachon University, Incheon, Korea.; 4 Department of Medicine, Institute of Biomedical Science and Technology, Konkuk University School of Medicine, Seoul, Korea.; 5 Department of Medical Education, Konkuk University School of Medicine, Seoul, Korea.

**Keywords:** Microglia, Neuropathic pain, Pregabalin, Allodynia, Cytokine

## Abstract

This study investigated the effects of pregabalin on microglial differentiation in rats with neuropathic pain (NP) induced by sciatic nerve ligation and transection. After confirming NP, the rats were randomly allocated to either a pregabalin or control group. The pregabalin group received intraperitoneal injections of 10 mg/kg pregabalin, while the control group received an equivalent volume of normal saline following surgery. On postoperative day 28, neuronal damage, microglial activity, and microglial differentiation were assessed. The pregabalin group exhibited significantly less neuronal damage compared to the control group, along with a significant decrease in activated microglial expression in both the brain and spinal cord. Pregabalin treatment also significantly altered the microglial phenotype expression, with a decrease in the M1 phenotype percentage and an increase in the M2 phenotype percentage in both the brain (M1 phenotype: 43.52 ± 12.16% and 18.00 ± 8.57% in the control and pregabalin groups, respectively; difference: 27.26 [15.18-42.10], p = 0.002; M2 phenotype: 16.88 ± 6.47% and 39.63 ± 5.82% in the control and pregabalin groups, respectively; difference 22.04 [17.17-32.70], p < 0.001) and the spinal cord ipsilateral to nerve injury (M1 phenotype: 44.35 ± 12.12% and 13.78 ± 5.39% in the control and pregabalin groups, respectively; difference 30.46 [21.73-44.45], p < 0.001; M2 phenotype: 7.64 ± 3.91% and 33.66 ± 7.95% in the control and pregabalin groups, respectively; difference 27.41 [21.21-36.30], p < 0.001). Overall, pregabalin treatment significantly decreased the microglial M1 phenotype while increasing the microglial M2 phenotype in NP rats.

## Introduction

The International Association for the Study of Pain (IASP) defines neuropathic pain (NP) as “pain caused by a lesion or disease of the somatosensory nervous system” 1. NP is characterised by hyperalgesia and allodynia 2. Despite vigorous efforts to clarify the mechanism of NP, understanding remains limited, and treatment remains challenging 3. Nevertheless, there have been endeavours to develop pharmacological therapies targeting specific aspects of NP. These efforts have yielded encouraging results, demonstrating improvements in the quality of life of NP patients. Pregabalin, also known as (S)-3-aminomethyl-5-methylhexanoic acid, is a pharmacological agent with specific NP targets. It is the pharmacologically active S-enantiomer of the racemic 3-isobutyl gamma amino butyric acid (GABA) analogue, known for its high binding affinity to the α2δ-1-containing voltage-gated calcium (Ca2+) channel (VGCC) within the nervous system 4. Pregabalin binds to α2δ-1-containing VGCC and modulates the release of various neurotransmitters, including glutamate, substance P, norepinephrine, and calcitonin gene-related peptide (CGRP) 5, resulting in symptomatic improvement. It is currently recommended as the first-line pharmacological agent for NP treatment.

In recent decades, microglia have been suggested to play a critical role in NP 6-8. Microglia are quiescent immune cells of the nervous system that are activated by molecules released from neurons following nerve injury 9. Activated microglia differentiate into distinct phenotypes, adopting either a pro-inflammatory (M1) or an anti-inflammatory (M2) phenotype.

Despite a detailed understanding of the molecular mechanisms of pregabalin in NP, its effect on the differentiation of activated microglia has not been investigated. Considering the association between microglial activation and differentiation, the inflammatory processes involved in NP occurrence 10, and the correlation between inflammation severity and NP signs and symptoms 11, pregabalin may influence microglial activation, differentiation, and inflammation in NP.

We hypothesized that pregabalin treatment for NP might modulate the M1 and M2 microglial phenotypes. Therefore, we assessed the effects of pregabalin treatment on the differentiation of these microglial phenotypes in NP rats.

## Materials and Methods

### Ethics

All experiments were performed in accordance with the National Institutes of Health (NIH) guidelines for the Care and Use of Laboratory Animals. After obtaining approval from the Institutional Animal Care and Use Committee (IACUC) of the Konkuk University, Seoul, Korea (approval number: KU21103), all experiments were conducted at the Konkuk University Laboratory Animal Research Centre in accordance with the IACUC guidelines.

### Animals 12

Male Sprague-Dawley (SD) rats aged 6-8 weeks and weighing 150-250 g were purchased from Orient Bio (Seongnam, Korea). The rats were housed individually in cages with ad libitum access to food and water. The room was maintained at a standard 12 hour light-dark cycle, with lights on at 7:00 and off at 19:00, and a temperature of 25°C. The rats were allowed to acclimate to these conditions for 7 days before NP induction surgery 12. All experimental procedures were performed during daytime at 13:00.

Prior to NP induction surgery, mechanical, and cold allodynia were assessed in all rats. Surgery was performed only if no mechanical or cold allodynia was detected. NP extent was evaluated at 3, 7, and 14 days after the surgery using the same techniques as before surgery. Rats were excluded if the NP expression during assessment (mechanical or cold allodynia) did not reach within 10% of pre-surgery levels. After confirming NP induction, the rats were randomly allocated into pregabalin and control groups. At 15, 21, and 28 days after the surgery, pregabalin rats received intraperitoneal administration of the drug (Lyrica®; Viatris Inc., Canonsburg, PA, USA) at a dosage of 10 mg/kg, mixed with 0.5 mL normal saline. Equal volumes of normal saline were administered to the control group. NP assessment was conducted before and 60 min after pregabalin or normal saline administration.

### NP assessment

NP was assessed by an observer blinded to group allocation, evaluating mechanical and cold allodynia using von Frey filaments and dry ice, respectively. Mechanical allodynia was assessed using von Frey filaments with forces of 0.6, 1.0, 1.4, 2.0, 4.0, 6.0, 8.0, 11.0, and 15.0 g. The rats were acclimated in a transparent test cage with a wire mesh metal floor for 30 min before the test. The rigid tip of the von Frey filament was applied perpendicularly to the skin of the lateral plantar area of the left hind paw until bending occurred. The test was initiated at a force of 2.0 g and if a response, including paw withdrawal or licking, occurred, weaker forces were applied using the Dixon up-down method 13-15. Conversely, if no response was elicited, stronger forces were applied using the same method. A maximum force of 15.0 g was taken as the threshold value.

Cold allodynia was assessed using dry ice at -80°C. The rats were placed in a transparent cage with a glass floor for 30 min for environmental acclimation. Dry ice powder was packed into a 10 mL syringe, compressed as much as possible, and then expelled through the syringe plunger, creating a pellet positioned 20-30 mm from the syringe tip. The pellets were gently and firmly applied to the glass beneath the hind paws, and the withdrawal response time, from initial contact to the hind foot lifting off the dry ice due to cold sensation, was measured.

### NP surgery

Surgery for NP was established on the basis of previous studies 12, 16. Anaesthesia was induced using 5% isoflurane (JW Pharmaceutical, Korea) administered via a mixture of oxygen at 300 mL/min and nitrous oxide at 700 mL/min. Once anaesthesia was achieved, the rats were transferred to the surgical platform, and anaesthesia depth was confirmed by pinching the hind foot. With the rat in a supine position, the tongue was gently pulled out using forceps. Subsequently, intubation was performed using a 1.77-inch-long, 16-gauge, 4.5 cm catheter (BD, Franklin Lakes, NJ, USA), and proper placement was verified by assessing symmetric chest expansion. The intubation catheter was connected to a ventilator (Harvard Apparatus, Holliston, MA, USA) configured to the following parameters: fraction of inspired oxygen (FiO2) of 0.5; inspiratory flow rate of 170 mL/min; tidal volume of 6 mL/kg; and respiratory rate of 80 breaths/min. Anaesthesia was maintained using isoflurane through the intubation catheter, regulated using a 4.0% vaporiser. Following intubation and ventilator setup, the rats were gently repositioned in the prone position, and intubation catheter depth was reassessed. Surgery was performed after proper positioning was confirmed. The left leg of each rat was secured to the platform using tape, and the hair around the left thigh was shaved. After sterilisation with 70% alcohol, a 2 cm incision was made along the posterior side of the heel to expose the sciatic nerve, including its three branches: the common peroneal, tibial, and sural nerves. Except for the sural nerve, these nerves were ligated using silk 5.0 and transected 2 mm distal to the ligation site. Following transection, the muscle and skin were sutured, and the incision site was disinfected.

### Brain and spinal cord preparation

Following the final NP assessment, conducted 60 min after pregabalin or normal saline administration, euthanasia was performed using isoflurane anaesthesia. The rats were placed in an anaesthesia induction chamber, where anaesthesia was induced using 5% isoflurane and maintained at 3% isoflurane. This anaesthesia was delivered via a gas mixture of oxygen at 300 mL/min and nitrous oxide at 700 mL/min. During anaesthesia, the rats were dissected, and the right atrium was exposed. Whole blood exsanguination was achieved by administering 1X phosphate-buffered saline (PBS) into the right atrium. Then the brain and the L5 spinal cord segment were dissected and secured. The L5 spinal cord segment was prepared for confirmation of neuronal damage, activated microglia with phenotypic differentiation, and degree of inflammation. The dissected L5 spinal cord segment and the right hemisphere at the thalamus level were fixed in a 4% paraformaldehyde solution for 24 h and individually submerged in 30% sucrose for 3 days in conical tubes. Subsequently, they were embedded in an optical cutting temperature compound and fixed in a plastic mould. After fixation, they were stored in a deep freezer at -80℃. Then the tissues were sectioned using a cryotome to achieve a thickness of 20 μm. Neuronal damage was assessed using terminal deoxynucleotidyl transferase (TdT) deoxyuridine triphosphate nick-end labelling (TUNEL) staining, while phenotypic differentiation of activated microglia was evaluated using immunofluorescence staining. TUNEL and microglia were analysed using an upright microscope and quantified using Image J software (NIH).

### TUNEL staining for neuronal damage in the brain and the spinal cord

TUNEL staining was performed to detect apoptotic neurons following the manufacturer's instructions (Cat No. G3250; Promega Corp., Madison, WI, USA). Tissue slides underwent permeabilisation with proteinase K solution (20 μg/mL) from the TUNEL staining kit for 10 min at room temperature. Subsequently, the tissues were washed with 1X PBS for 5 min. Following washing, 100 µL equilibration buffer was gently applied onto the tissues, and they were incubated for 10 min at room temperature. Then the tissues were stained with TdT buffer to detect neuronal damage. The TdT buffer consisted of a mixture of 10% nucleotide mix (5 μL) and 2% TdT solution (1 μL) diluted in equilibration buffer (45 μL). The tissues were immersed in 50 µL TdT buffer for 60 min at room temperature. For the negative control, TdT buffer was prepared using distilled water instead of the TdT solution. After labelling with the TdT buffer, the tissues were washed with 1X PBS for 5 min. Subsequently, the tissues were stained with 4,6-diamidino-2-phenylindole (DAPI) solution for nuclear staining. After staining, the tissues were washed with 1X PBS for 5 min, and anti-fade medium was added. Cover slips were placed over the tissues and sealed with nail polish. Neuronal damage was analysed using an upright microscope (BX 61; Olympus, Japan). The percentage of cells with positive TUNEL staining among those with positive DAPI staining was determined.

### Activated microglia in the brain and the spinal cord

Double immunofluorescence staining was performed to detect activated microglia. The tissues were immersed in 5% normal goat serum (CAT No. ab7481; Abcam Ltd., Cambridge, UK) for 1 h at room temperature to block nonspecific binding. After blocking, the tissues were washed with 1X PBS for 5 min and stained with rabbit transmembrane protein (TMEM)-119 (CAT No. NBP2-30551; Novus Biologicals, Minneapolis, MN, USA) and mouse ionized calcium-binding adaptor (Iba1) (CAT No. MA5-27726; Invitrogen, Waltham, MA, USA) in 1% normal goat serum for 1 h at room temperature. Following staining, rabbit immunoglobulin G (CAT No. bs-0295P; Bioss Antibodies, Woburn, MA, USA) and mouse immunoglobulin G (CAT No. A56854; Thermo Fisher Scientific, Waltham, MA, USA) were added for 1 h. Then the tissues were washed three times with 1X PBS. Subsequently, the tissues were incubated with goat Alexa Fluor 488 (CAT No. A-11008; Thermo Fisher Scientific) and goat Alexa Fluor 594 (CAT No. A-11012; Thermo Fisher Scientific) secondary antibodies in 1% normal goat serum for 1 h at room temperature. After staining, the tissues were washed three times with 1X PBS. Then DAPI nuclear staining was performed in the dark for 5 min at room temperature. Following DAPI staining, the tissues were washed with 1X PBS for 5 min, and anti-fade medium was added. Cover slips were placed over the tissues and sealed with nail polish. Activated microglia were analysed under an upright microscope (BX 61; Olympus). The percentage of cells with positive TMEM 119 and Iba1 staining among those with positive DAPI staining was determined.

### Phenotypic differentiation of microglia in the brain and spinal cord

Double immunofluorescence staining was conducted using a similar procedure as that used for detecting activated microglia. However, different primary antibodies were used to identify M1 and M2 phenotypes. For the M1 phenotype, rabbit cluster of differentiation (CD)-16 (CAT No. bsm-54679R; Bioss Antibodies) and mouse Iba1 were used as primary antibodies. For the M2 phenotype, rabbit CD206 (CAT No. bs-4727R; Bioss Antibodies) and mouse Iba1 were used. CD16-positive cells among those with positive DAPI were defined as the M1 phenotype and expressed as a percentage. Similarly, CD206-positive cells among those with positive DAPI were defined as the M2 phenotype.

### Brain and spinal cord cytokines

The tissues from the right hemisphere and the left side of the L5 spinal cord segment were homogenized using cold 1X PBS and centrifuged at 12,000 g for 15 min at 4°C. The resulting supernatants were collected into Eppendorf tubes for the ELISA assay, performed according to the manufacturer's instructions. The ELISA assay assessed the levels of pro-inflammatory cytokines, tumour necrosis factor (TNF)-α (CAT No. KRC3011; Invitrogen) and interleukin (IL)-1β (CAT No. RLB00, R&D Systems, Minneapolis, MN, USA), as well as anti-inflammatory cytokines IL-4 (CAT No. BMS628; Invitrogen) and IL-10 (CAT No. BMS629; Invitrogen). Prior to ELISA, total protein levels were measured using the Bradford assay method following the manufacturer's instructions. Results from different samples were obtained for those with equivalent protein concentrations.

### Statistics

The primary outcome was the microglial phenotypic expression (M1 or M2) on the ipsilateral side of the spinal cord following sacrifice. A pilot study involving three rats per group indicated that the M1 phenotypes in the spinal cord accounted for 42.14 ± 15.48% and 18.12 ± 3.52% of the control and pregabalin groups, respectively. Meanwhile, the M2 phenotypes accounted for 9.27 ± 2.60% and 41.18 ± 19.25%, respectively. Based on the pilot study, a sample size of six rats for the M1 phenotype expression and six for the M2 phenotype expression was calculated to achieve a power of 0.9 and an α of 0.05.

Intragroup and intergroup differences were analysed using an unpaired t-test and two-way repeated analysis of variance (ANOVA), with an F value for intergroup differences, in GraphPad Prism software (v. 10.0; GraphPad Software Inc., Boston, MA, USA). Post hoc testing for multiple comparisons involved Tukey's multiple comparisons test, and adjusted p-values are reported. A p-value < 0.05 was considered significant. Data are presented as means ± standard deviations.

## Results

Twelve rats were enrolled in the study and evenly allocated into two groups. NP-inducing surgery was successfully performed in all experiments without any complications. Pregabalin or normal saline was administered without adverse events.

**Figure 1:**
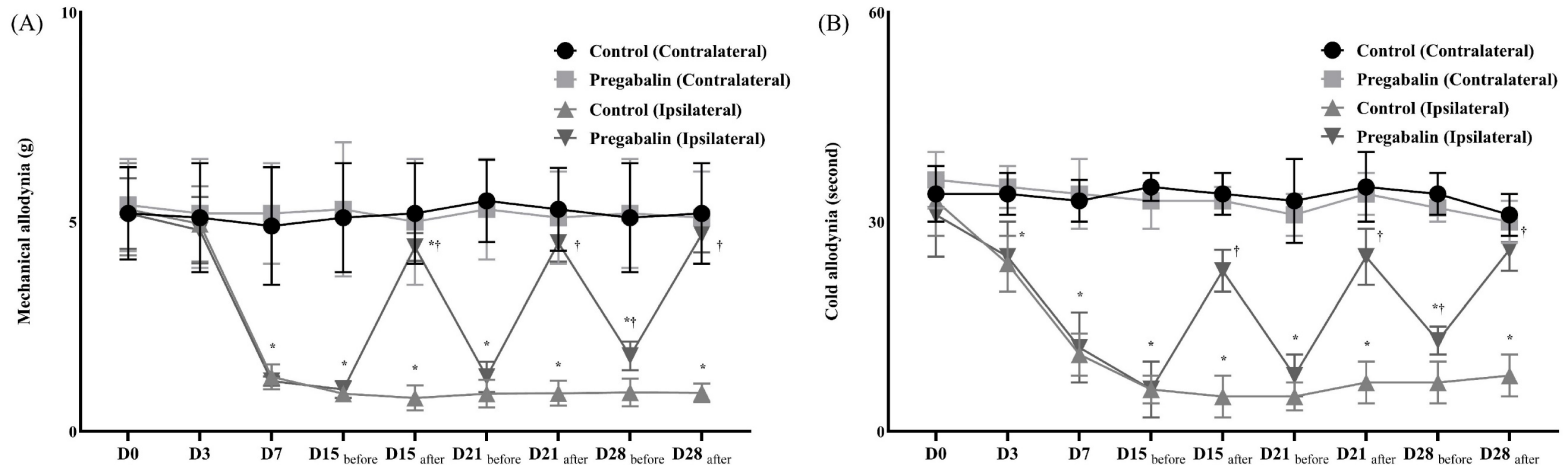
Assessment of mechanical (A) and cold (B) allodynia for neuropathic pain before and after the treatment of normal saline or pregabalin. (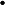) Contralateral side for the surgery, Control group, (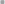) Contralateral side for the surgery, Pregabalin group, () Ipsilateral side for the surgery, Control group, () Ipsilateral side for the surgery, Pregabalin group. **Abbreviations:** Control, Control group; Pregabalin, Pregabalin group; D0, before the surgery; D, day after the surgery; before, before the treatment of normal saline or pregabalin; after, 60 minutes after the treatment of normal saline or pregabalin. ^*^p < 0.05 compared with D0 in intra-group variance. ^†^*p* < 0.05 compared with Control group.

The animal NP model was successfully established in both groups without complications. However, the pregabalin group exhibited significantly lower sensitivity to allodynia, including mechanical and cold stimuli, at the NP leg compared to the control group (Figure 1). Before surgery, there were no differences in withdrawal response to mechanical and cold allodynia between the contralateral and ipsilateral sides in both groups. However, after surgery, the ipsilateral side in both groups showed significantly lower thresholds for mechanical stimulation and an earlier withdrawal response to cold stimulation compared to the contralateral side. Following pregabalin or normal saline treatment on days 15, 21, and 28 after the surgery, the withdrawal response to von Frey filaments and dry ice on the contralateral side showed no significant changes in the two groups. However, on the ipsilateral side, responses to mechanical and cold stimulation were lower in the pregabalin group compared to the control group.

**Figure 2:**
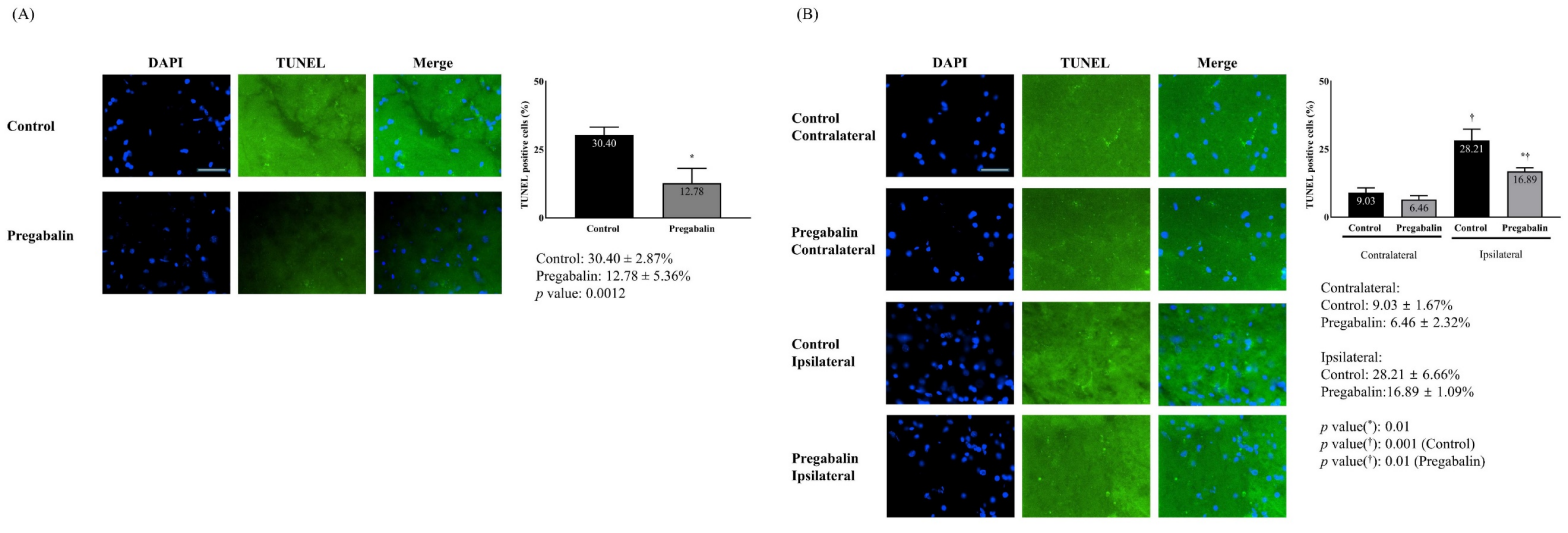
Neuronal damage in the brain (A) and the L5 segment of the spinal cord (B). **Abbreviations:** DAPI, 4,6-diamidino-2-phenylindole staining; terminal deoxynucleotidyl transferase (TdT) deoxyuridine triphosphates (dUTP) nick end labeling (TUNEL) staining; Merged, merged image of DAPI staining and TUNEL staining. Control, Control group; Pregabalin, Pregabalin group; Contralateral, contralateral side for surgery; Ipsilateral, Ipsilateral side for the surgery. ^*^p < 0.05 compared with Control group. ^†^*p* < 0.05 compared with contralateral side in each group.

The extent of neuronal damage in the brain, assessed via TUNEL staining, was significantly reduced in the pregabalin group compared to the control group (12.78 ± 5.36% vs. 30.40 ± 2.87%, difference: 16.4% [14.06-18.55], p = 0.001) (Figure 2). Neuronal damage at the ipsilateral spinal cord following surgery was significantly greater than at the contralateral side in both groups (28.21 ± 6.66% vs. 9.03 ± 1.67%, difference: 20.45% [15.76-23.54], p = 0.001 for the control group; 16.89 ± 1.09% vs. 6.46 ± 2.32%, difference: 10.25% [9.85-12.35], p = 0.010 for the pregabalin group). However, neuronal damage at the ipsilateral spinal cord was significantly lower in the pregabalin group compared to the control group (p = 0.010; Figure 2).

**Figure 3:**
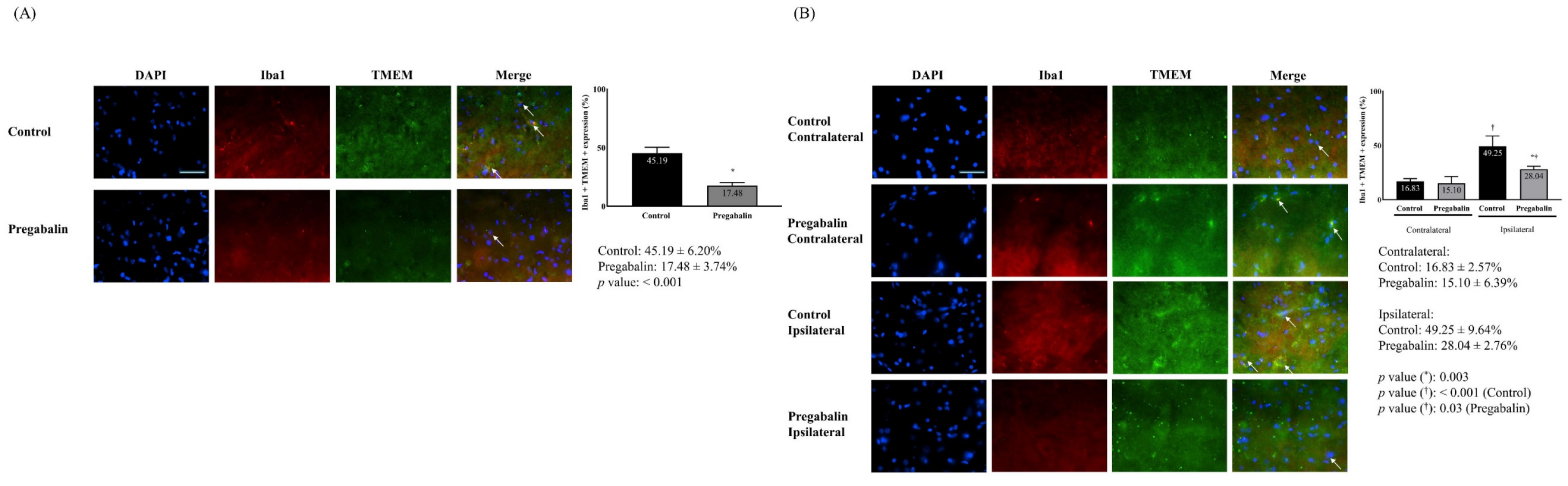
Activated microglia in the brain (A) and the L5 segment of the spinal cord (B). **Abbreviations:** DAPI, 4,6-diamidino-2-phenylindole staining; Iba1, ionized calcium-binding adapter molecule 1 staining; TMEM, rabbit transmembrane protein 119 staining; Merged, merged image of DAPI staining, Iba1 staining and TMEM staining; Control, Control group; Pregabalin, Pregabalin group; Contralateral, contralateral side for surgery; Ipsilateral, Ipsilateral side for the surgery. ^*^p < 0.05 compared with Control group. ^†^*p* < 0.05 compared with contralateral side in each group.

Activated microglia in the brain and spinal cord exhibited similar patterns as for neuronal damage. The pregabalin group showed a significantly lower increase in activated microglia in the brain. The ipsilateral side exhibited significantly increased microglial activation following surgery compared to the contralateral side in both groups (17.48 ± 3.74% and 45.19 ± 6.20% for the pregabalin and control groups, respectively; difference: 25.46% [19.45-33.43], p < 0.001 in the brain; 49.25 ± 9.64% at the ipsilateral side vs. 16.83 ± 2.57% at the contralateral side, difference: 30.75% [24.45-41.57], p < 0.001 in the control group; 28.04 ± 2.76% at the ipsilateral side vs. 15.10 ± 6.39% at the contralateral side, difference: 15.46% [7.54-20.67], p = 0.030 in the pregabalin group). Microglial activation at the ipsilateral spinal cord was significantly lower in the pregabalin group than in the control group (p = 0.003; Figure 3).

**Figure 4:**
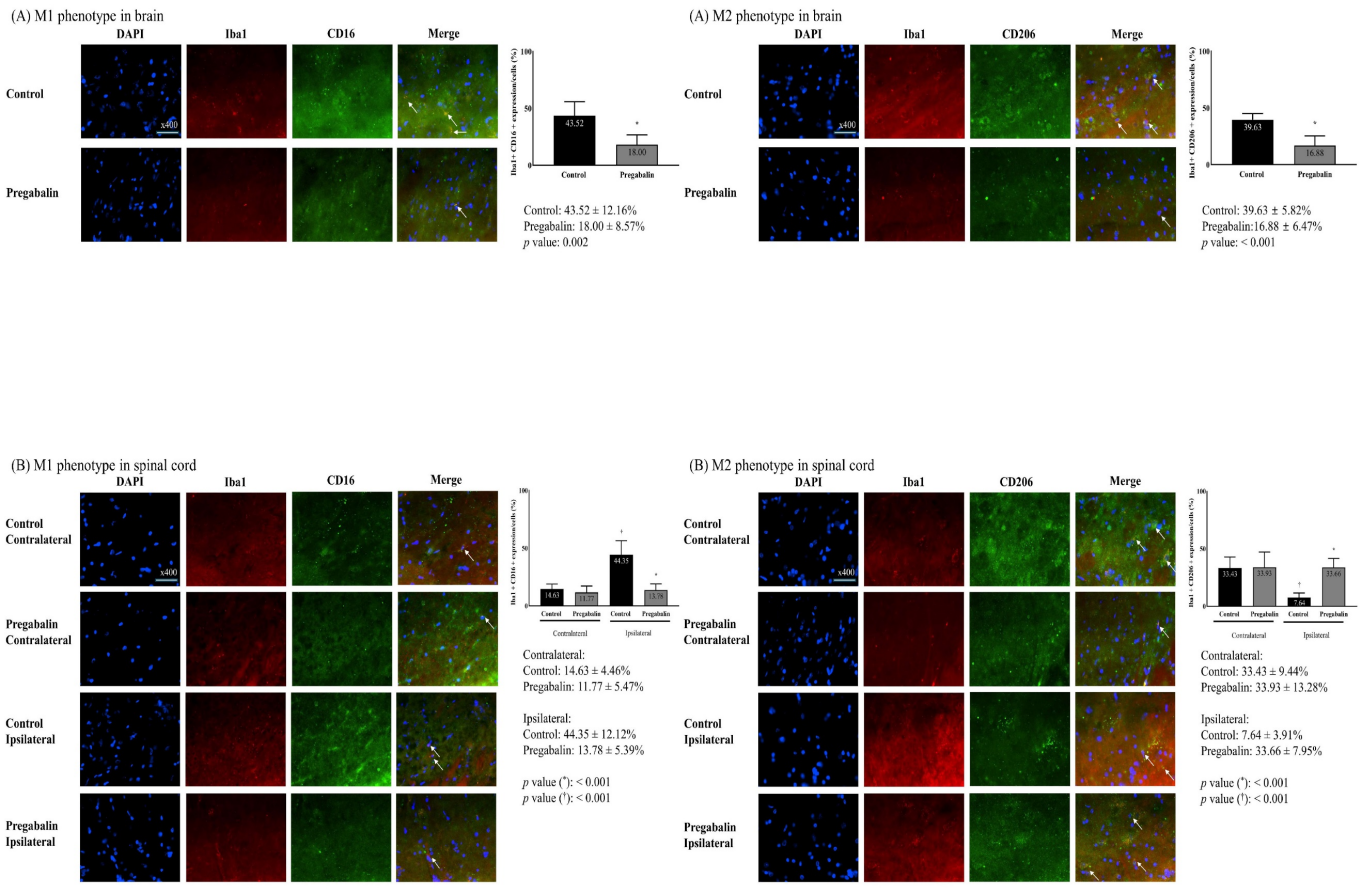
Differentiation of microglia in the brain (A) and the L5 segment of the spinal cord (B). **Abbreviations:** DAPI, 4,6-diamidino-2-phenylindole staining; Iba1, ionized calcium-binding adapter molecule 1 staining; CD, cluster differentiation; Merged, merged image of DAPI staining, Iba1 staining, and CD16 (for M1 phenotype) or CD206 staining (for M2 phenotype); Control, Control group; Pregabalin, Pregabalin group; Contralateral, contralateral side for surgery; Ipsilateral, Ipsilateral side for the surgery. ^*^p < 0.05 compared with Control group. ^†^*p* < 0.05 compared with contralateral side in each group.

Microglial differentiation into the M1 and M2 phenotypes in the brain revealed significant differences. A significantly lower M1 phenotype expression and a significantly higher M2 phenotype expression were observed in the pregabalin group compared to the control group (M1 phenotype: 43.52 ± 12.16% and 18.00 ± 8.57% in the control and pregabalin groups, respectively; difference: 27.26% [15.18-42.10], p = 0.002; M2 phenotype: 16.88 ± 6.47% and 39.63 ± 5.82% in the control and pregabalin groups, respectively; difference: 22.04% [17.17-32.70], p < 0.001) (Figure 4). The difference was statistically significant (F1,10 = 17.37, p < 0.01).

The M1 phenotype at the spinal cord in the pregabalin group did not exhibit any significant change (11.77 ± 5.47% at the contralateral side vs. 13.78 ± 5.39% at the ipsilateral side, p = 0.530). Compared to the pregabalin group, the control group showed a significantly higher M1 phenotype expression at the spinal cord ipsilateral to nerve injury compared to the contralateral side (14.63 ± 4.46% at the contralateral side vs. 44.35 ± 12.12% at the ipsilateral side, difference: 28.86% [20.45-43.50], p < 0.001). The difference was statistically significant (F1,10 = 23.273, p < 0.01). Moreover, the M1 phenotype at the ipsilateral spinal cord was significantly lower in the pregabalin group than in the control group (p < 0.001; Figure 4).

The M2 phenotype percentage of the spinal cord in the pregabalin group did not exhibit any significant changes (33.93 ± 13.28% at the contralateral side vs. 33.66 ± 7.95% at the ipsilateral side, p = 0.970). Compared to the pregabalin group, the control group showed a significantly lower M2 phenotype expression percentage at the spinal cord ipsilateral to nerve injury, than at the contralateral side (33.43 ± 9.44% at the contralateral side vs. 7.64 ± 3.91% at the ipsilateral side, difference: 27.15% [21.65-35.58], p < 0.001). The difference was statistically significant (F1,10 = 19.643, p < 0.01). Moreover, the M2 phenotype percentage at the ipsilateral spinal cord was significantly higher in the pregabalin group than in the control group (p < 0.001; Figure 4).

The pregabalin group exhibited a significantly lower expression of pro-inflammatory cytokines, including TNF-α and IL-1β, and a significantly higher expression of anti-inflammatory cytokines, including IL-4 and IL-10, compared to the control group (Table 1).

## Discussion

The present study showed that pregabalin improved mechanical and cold allodynia in rats with NP. Moreover, pregabalin significantly attenuated the neuronal damage in the brain and the spinal cord of rats with NP. Administering pregabalin to rats with NP, relative to levels in control tissues from animals untreated, induced a significant difference in microglial phenotype expression, decreasing the M1 phenotype and increasing the M2 phenotype in both brain and spinal cord tissues.

Gabapentin and pregabalin, as anticonvulsants targeting VGCC, are widely used in NP management as first-line treatments. Pregabalin offers the advantages of faster absorption, rapid action, and fewer side effects. Therefore, although gabapentin has more often been used in previous studies, we used pregabalin to investigate NP. Several studies have demonstrated the superior efficacy of pregabalin over gabapentin in animal NP models 17, 18.

Microglia are central nervous system macrophages that are swiftly activated under pathological conditions. However, their role in resting conditions remains poorly understood. Microglial activation is accompanied by morphological changes, upregulation of surface antigens, and secretion of cytotoxic and neurotrophic molecules 19, 20. Although the precise role of microglia in NP remains poorly understood, their activation has been implicated in NP pathogenesis 21-23. Recent studies have identified microglia as important therapeutic targets for NP 24, with several studies demonstrating that inhibiting activated microglia and shifting from the M1 to M2 phenotype is associated with NP hyperalgesia alleviation 25, 26.

In our study, the specific pathway or factor underlying the changes in microglial differentiation after pregabalin treatment in rats could not be determined. However, we hypothesised that the mechanism was evidence-based. Thrombospondin-4 (TSP4), an extracellular matrix glycoprotein originating from astrocytes and microglia, plays a mediating role in cell-matrix interactions 27, 28. It has been implicated in NP pathogenesis 29, 30, with upregulated TSP4 secretion by astrocytes and glial cells observed following nerve damage in NP. This upregulation results in binding to α2δ-1-containing VGCCs, promoting excitatory synaptogenesis in the nervous system 31, enhancing microglial accumulation, and facilitating differentiation into the M1 phenotype 32. Therefore, when pregabalin binds to α2δ-1-containing VGCCs, it may inhibit TSP4 binding, thereby reducing macrophage differentiation into the M1 phenotype, as observed in the present study.

Our findings regarding microglial differentiation were supported by changes in cytokine levels. Pregabalin treatment in NP rats significantly decreased pro-inflammatory cytokines, including TNF-α and IL-1β, and increased anti-inflammatory cytokines, including IL-4 and IL-10.

Previous research has reported prominent expressions of transcripts encoding pro-inflammatory cytokines, including TNF-α, IL-1β, and IL-6, during NP onset 33. Microglia, activated by stressors, were found to release TNF-α and IL-1β through the p38 mitogen activated protein kinase (MAPK) pathway, but the microglial phenotype was not defined in the previous studies 34. MAPK inhibition is associated with a reduction in activated microglia and alleviation of NP-related symptoms, including allodynia 34-36. Furthermore, pregabalin treatment has been demonstrated to induce IL-10 release from microglia, but not from astrocytes, leading to alleviation of allodynia and hyperalgesia 37. Consistent with these findings, Burke et al. reported a decrease in the M1 phenotype, IL-1β, and IL-6, along with an increase in the M2 phenotype and IL-10, contributing to symptom attenuation in NP 38.

In this study, we investigated neuronal damage and microglial activation in both the brain and spinal cord, focusing on phenotypic differentiation and inflammation degree. Remarkable changes were observed in the brain, indicating that signals from damaged peripheral nerves affected not only the spinal cord but also the brain 39, 40. These findings suggest that NP patients may experience not only neurological but also psychological issues, although this aspect was not specifically evaluated in our study.

It is important to note that, while pregabalin is commonly used to manage NP symptoms and signs, it does not recover damaged nerves. In our study, pregabalin administration improved mechanical and cold allodynia and reduced neural damage. Despite our significant findings, further studies are needed to determine the mechanism underlying this improvement in neural damage.

Several limitations to our study should be considered. First, the study involved male SD rats and although this has not been extensively explored, microglial responsiveness or expression may exhibit sex differences 41, 42. Sex-related differences in pain sensitivity have also been reported in several studies, although the underlying mechanism remains unclear 43, 44. Therefore, our findings may not be generalizable to female SD rats. However, the results provide the basis for a hypothesis that may merit a further clinical investigation using biological probes, although the study cannot be generalized to a clinical situation. Second, NP was induced through nerve ligation and transection, which have been shown to produce similar neurological manifestations in rats 45, 46. However, the magnitude of response to the same stimuli may differ depending on the method used for NP induction 45, 46. Third, the study did not include a sham group, which could have provided a more concrete basis for our conclusions. However, ethical considerations must be taken into account when conducting additional studies involving sham procedures.

In conclusion, pregabalin treatment significantly decreased the microglial M1 phenotype and increased the microglial M2 phenotype in NP rats. The observed improvement in allodynia following treatment may be associated with microglial differentiation.

## Figures and Tables

**Table 1:** Cytokines in the brain and the spinal cord.

	Brain			Spinal cord		
	Control groupMean (SD)	Pregabalin groupMean (SD)	*P* value	Control groupMean (SD)	Pregabalin groupMean (SD)	*P* value
Pro-inflammatory cytokines						
TNF-α (pg/ml)	2888.00 (426.40)	1332.00 (134.1)	0.038	1695.20 (132.31)	998.73 (95.23)	0.002
IL-1β (pg/ml)	503.90 (55.73)	126.30 (18.35)	0.012	367.65 (33.17)	137.13 (34.77)	0.004
Anti-inflammatory cytokines						
IL-4 (pg/ml)	4.25 (2.54)	18.50 (1.55)	0.021	2.44 (0.95)	9.78 (1.03)	0.012
IL-10 (pg/ml)	138.10 (17.70)	345.10 (47.34)	0.029	169.03 (6.85)	272.00 (16.76)	0.010

**Abbreviations:** TNF-α, tumor necrosis factor-α; IL, interleukin.
